# Intranasal Tat-modified PEG-PCL nanomicelles delivering anti-RelA siRNA attenuate ischemia-reperfusion injury

**DOI:** 10.1016/j.omtn.2026.103028

**Published:** 2026-07-21

**Authors:** Mitsuyoshi Fukuda, Takayuki Oguma, Taiki Nagatomo, Taiki Yamaguchi, Yasuhiro Kosuge, Toyofumi Suzuki, Takanori Kanazawa

**Affiliations:** 1 Laboratory of Pharmaceutics, School of Pharmacy, Nihon University, 7-7-1 Narashinodai, Funabashi, Chiba 274-8555, Japan; 2 Center for Clinical Research, Hamamatsu University Hospital, 1-20-1 Handayama, Chuo-ku, Hamamatsu, Shizuoka 431-3192, Japan; 3 Department of Clinical Pharmacology, Graduate School of Biomedical Sciences, Tokushima University, 1-78-1, Shoumachi, Tokushima 770-8505, Japan; 4 Laboratory of Pharmacology, School of Pharmacy, Nihon University, 7-7-1 Narashinodai, Funabashi, Chiba 274-8555, Japan

**Keywords:** MT: delivery strategies, nose-to-brain delivery, polymer micelle, siRNA, NF-κB, RelA, transient middle cerebral artery occlusion, neuroprotection, inflammation inhibition

## Abstract

Inflammatory responses centered on microglial activation are deeply involved in the progression of cerebral ischemia-reperfusion injury, and RelA, a subunit of nuclear factor κB (NF-κB), acts as a central mediator of these responses. In this study, we evaluated the efficacy of a nose-to-brain drug delivery system that combines intranasal administration with polymeric nanomicelles modified with the cell-penetrating peptide Tat (polyethylene glycol [PEG]-polycaprolactone [PCL]-Tat) to deliver RelA-targeting small interfering RNA (siRNA), referred to as siRelA. The PEG-PCL-Tat-siRelA complex exhibited efficient intracellular uptake by BV-2 microglial cells and significantly suppressed RelA mRNA expression. No significant cytotoxicity was observed in BV-2 or primary cultured mouse hippocampal neurons. In addition, the complex showed high siRNA stability in the cerebrospinal fluid. Using a radiolabeled model siRNA, intranasal administration of PEG-PCL-Tat to mice subjected to transient middle cerebral artery occlusion (t-MCAO) significantly enhanced siRNA distribution in ischemic brain regions. Intranasal administration of PEG-PCL-Tat-siRelA significantly reduced the infarct volume in ischemic brain regions of the t-MCAO model mice. Furthermore, suppression of RelA mRNA expression in ischemic brain tissue was accompanied by significant reductions in the mRNA expression of NF-κB-downstream inflammatory cytokines. Taken together, these results suggest that intranasal delivery of siRelA using PEG-PCL-Tat represents a promising novel neuroprotective therapeutic strategy mediated by the modulation of inflammatory responses.

## Graphical abstract

**Figure:**
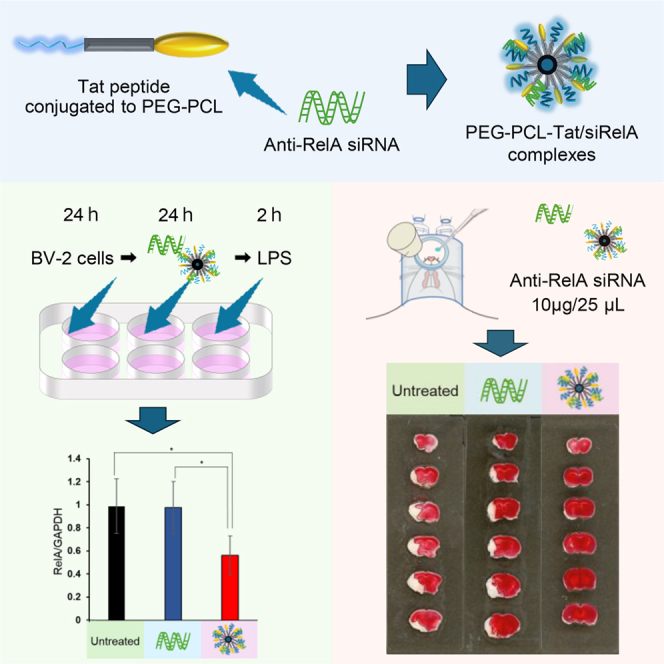

## Teaser

Kanazawa and colleagues demonstrate that intranasal Tat-modified PEG-PCL nanomicelles effectively deliver anti-RelA siRNA to ischemic brain regions, suppressing NF-κB-mediated inflammation and reducing infarct volume in MCAO mice. These findings highlight a promising non-invasive neuroprotective strategy for ischemia-reperfusion injury.

## Introduction

Stroke has emerged as a leading cause of death worldwide, following myocardial infarction and COVID-19, and it has been responsible for approximately seven million deaths annually in recent years.^1^ Stroke is broadly classified into three major types: ischemic, hemorrhagic, and subarachnoid. Ischemic stroke accounts for approximately 80% of all cases.^2^ Following the onset of cerebral ischemia, interruption of blood flow leads to progressive tissue damage, resulting in the formation of an infarct core with irreversible injury and a surrounding penumbral region consisting of potentially salvageable tissue.^3^ During the treatment of ischemic stroke, salvaging the penumbral region during the acute phase suppresses the progression of neuronal cell death, leading to improved survival and functional outcomes. The brain contains both neurons and glial cells, and expansion of the infarct core during ischemic stroke is mainly mediated by neuroinflammation involving the activation of microglia, a type of glial cell.^4^^,^^5^ Following cerebral ischemia, microglial-produced inflammatory cytokines trigger inflammatory responses in the brain. The upregulation of genes associated with these inflammatory responses has been reported not only in glial cells but also in neurons,^6^ indicating that neurons themselves are involved in the inflammatory response.

Neuronal cell necrosis caused by cerebral ischemic injury, such as ischemic stroke, is considered to involve inflammation mediated by nuclear factor κB (NF-κB).^7^ NF-κB regulates the transcription of genes involved in inflammatory responses, including tumor necrosis factor α (TNF-α), interleukin 1β (IL-1β), and interleukin 6 (IL-6).^8^ The NF-κB transcription factor family comprises five subunits: RelA, RelB, c-Rel, p50, and p52. These subunits function as transcription factors through the formation of homodimers or heterodimers.^9^^,^^10^ Among these, RelA-mediated signaling is deeply involved in post-ischemic inflammatory responses and is activated by stress stimuli such as TNF-α, IL-1β, and lipopolysaccharide (LPS). The inhibition of RelA-dependent inflammatory signaling is expected to suppress excessive inflammatory responses following cerebral ischemia and attenuate the progression of neuronal cell death, potentially leading to improved outcomes after ischemic stroke.

In clinical practice, tissue plasminogen activator (t-PA) therapy is used for thrombolysis during the hyperacute phase of cerebral infarction, thereby enabling salvage of the penumbral region by restoring blood flow. However, when t-PA administration is delayed, its beneficial effects on clinical outcomes diminish, and mortality increases. The effective treatment window is considered to be within 4.5 h of symptom onset.^11^ In addition, thrombolytic therapy has limited efficacy for large-vessel occlusions of the major cerebral arteries. Consequently, mechanical thrombectomy using stent retrievers or aspiration catheters has become widely adopted as a therapeutic option that may be considered within 24 h of symptom onset depending on patients’ clinical condition.^12^^,^^13^^,^^14^ Although these advances have improved recanalization rates in ischemic regions, they have not led to substantial improvements in functional recovery or the successful development of neuroprotective agents. In Japan, edaravone, a free-radical scavenger, has been approved as a neuroprotective drug for attenuating brain injury associated with cerebral ischemia and reperfusion. Nevertheless, its clinical efficacy remains insufficiently supported by robust evidence, and it has not been adopted as a standard therapy outside Japan.^15^^,^^16^ Novel therapeutic strategies capable of suppressing cerebral ischemia-reperfusion injury remain an unmet clinical need.

Recently, several nucleic acid therapeutics have been approved for the treatment of inherited neuromuscular and neurodegenerative disorders. These include patisiran,^17^ the world’s first small interfering RNA (siRNA) therapy approved for transthyretin-mediated familial amyloid polyneuropathy; viltolarsen,^18^ an antisense oligonucleotide for Duchenne muscular dystrophy; and nusinersen,^19^ an antisense oligonucleotide indicated for spinal muscular atrophy. Nucleic acid therapeutics have been reported to sequence specifically knockdown target molecules in the brain^20^^,^^21^^,^^22^ and are expected to be therapeutic agents for central nervous system disorders. In addition, the delivery of siRNAs to the brain ischemic injury sites is considered effective in suppressing the inflammatory cascade during cerebral ischemic injury, which is the target of this study. Nucleic acid therapeutics are water-soluble macromolecules susceptible to metabolic degradation, resulting in poor *in vivo* stability. Their large molecular size prevents them from crossing the blood-brain barrier (BBB) and blood-cerebrospinal fluid barrier (BCSFB), which strictly limits their access to the brain via systemic circulation, such as intravenous administration.^23^ Low cellular uptake of nucleic acid therapeutics remains a challenge. Drug delivery systems that enhance brain delivery are being actively investigated for the treatment of brain diseases.

Nasal administration has attracted attention as a route for delivering medium- and high-molecular-weight therapeutics into the brain, where BBB penetration is not an issue because it involves a unique pathway known as the nose-to-brain delivery route that enables direct transport of drugs to the brain without passing through the systemic circulation.^24^^,^^25^^,^^26^^,^^27^ This route is considered a potentially effective means of delivering siRNAs to the brain.^28^ Intranasal administration is a simple and non-invasive approach that enables rapid drug administration, even in the hyperacute phase of cerebral infarction or in patients with dysphagia. However, intranasal administration has several limitations, including low permeability across the nasal mucosa and rapid drug loss due to mucociliary clearance, which make its clinical application challenging. Recent studies have indicated that nanocarriers enhance drug stability and nasal mucosal permeability, thereby improving drug transport to the brain via the nose-to-brain delivery pathway.^29^^,^^30^^,^^31^ We previously reported that cell-penetrating peptide-modified polymeric micelles (PEG-PCL-Tat) can help overcome key challenges associated with intranasal administration, including limited nasal mucosal permeability and mucociliary clearance.^32^^,^^33^ PEG-PCL-Tat is a polymeric nanomicelle in which the cell-penetrating peptide Tat chemically conjugated to a block co-polymer (PEG-PCL) composed of hydrophilic polyethylene glycol (PEG) and hydrophobic polycaprolactone (PCL) forms a core.^34^ The combination of PEG-PCL-Tat with intranasal administration may represent a therapeutic strategy capable of suppressing the inflammation associated with cerebral infarction and providing neuroprotective effects.

This study evaluated the brain delivery and therapeutic efficacy of the nose-to-brain route with PEG-PCL-Tat and anti-RelA siRNA (siRelA) targeting RelA, a subunit of NF-κB, for the treatment of cerebral infarction. We examined siRNA stability in the cerebrospinal fluid (CSF), the toxicity of PEG-PCL-Tat, siRNA uptake, and RelA mRNA knockdown in microglial cells and primary cultured mouse hippocampal neurons. In a mouse model of transient middle cerebral artery occlusion (t-MCAO), we assessed the distribution of the siRelA-PEG-PCL-Tat complex to both the infarcted ipsilateral and untreated contralateral forebrain regions, as well as its effects on infarct volume and the expression of RelA and other inflammation-related mRNAs following intranasal administration.

## Results

### Physicochemical characterization and stability of PEG-PCL-Tat-siRNA

The physicochemical properties of the nanomicelles prepared with varying nitrogen-to-phosphate (N/P) ratios of the PEG-PCL-Tat-siRelA complex (5–30) are listed in Table 1. The particle size ranged from 60 to 110 nm (diameter), and the zeta potential was positive at all N/P ratios, ranging from 5 to 20 mV. As the N/P ratio increased, the particle size decreased, and the zeta potential increased. The stability of the PEG-PCL-Tat-siRelA complex prepared at an N/P ratio of 30 was evaluated in the CSF over time. The complex exhibited almost no degradation, even after 24 h of incubation in CSF (Figure 1). In contrast, naked siRelA showed a residual fraction below 60% after 2 h, with more than a half degradation after 24 h.

**Table 1:** Mean particle diameter and zeta potential of PEG-PCL-Tat-siRelA complexes

N/P ratio	Mean particle diameter (nm)	Zeta potential (mV)
5	111.8 ± 2.6	+5.53 ± 2.8
10	98.0 ± 3.0	+8.93 ± 2.1
20	74.2 ± 1.9	+16.07 ± 0.6
30	60.9 ± 1.7	+20.39 ± 2.1

Particle size and zeta potential values represent the mean ± SD (*n* = 3). N/P ratio, nitrogen-to-phosphate ratio.

**Figure 1:**
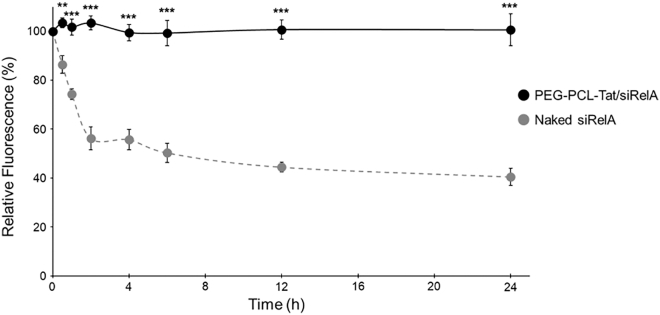
Stability of PEG-PCL-Tat-siRelA complexes in mouse cerebrospinal fluid The PEG-PCL-Tat-siRelA complexes (N/P ratio = 30) were prepared by incubation at room temperature for 30 min. Mouse cerebrospinal fluid (CSF; 10 μL per 1 μg siRNA) was added to naked siRNA and PEG-PCL-Tat-siRelA complexes (N/P ratio = 30), followed by incubation at room temperature. The siRNAs were decondensed by adding 0.05 mM dextran sulfate and stained with SYBR Green. Each value represents the mean ± SD (*n* = 3). Statistical analysis was performed using Student’s *t* test. ∗∗*p* < 0.01, ∗∗∗*p* < 0.001.

### Cellular uptake efficiency of PEG-PCL-Tat nanomicelles in BV-2 cells

The uptake of naked fluorescent 6-carboxyfluorescein-aminohexylphosphoramidite (FAM)-siRNA and PEG-PCL-Tat-FAM-siRNA at various N/P ratios by the immortalized mouse microglial (BV-2) cells is shown in Figures 2 and S1. Naked FAM-siRNA was barely internalized by the cells. In comparison, PEG-PCL-Tat-FAM-siRNA was taken up by cells across all N/P ratios, with uptake increasing as the N/P ratio increased. Notably, nanomicelles with an N/P ratio of 30 showed approximately 20% cellular uptake.

**Figure 2:**
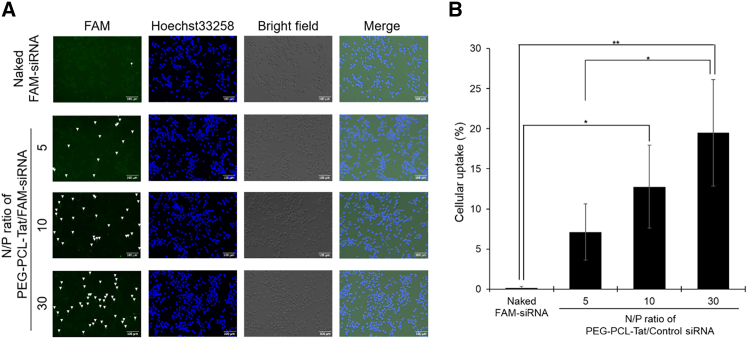
Cellular uptake of PEG-PCL-Tat/siRNA complexes in BV-2 cells (A) Representative fluorescence microscopy images of BV-2 cells after 4 h incubation with naked FAM-siRNA or PEG-PCL-Tat-FAM-siRNA complexes at N/P ratios of 5, 10, and 30. Green fluorescence indicates FAM-siRNA, and nuclei were counterstained with Hoechst 33258 (blue). White arrowheads indicate representative intracellular FAM-siRNA signals. Merged images are shown in the right; scale bars, 100 μm. (B) Quantitative analysis of cellular uptake. The percentage of FAM-positive cells relative to the total number of Hoechst-stained nuclei was calculated from fluorescence images, using a cell-counting method within the same fields. Data are presented as the mean ± SD (*n* = 3). Statistical analysis was performed using one-way ANOVA followed by Tukey’s post hoc test. ∗*p* < 0.05, ∗∗*p* < 0.01.

### Knockdown by PEG-PCL-Tat-siRelA in BV-2 cells

Naked siRelA, PEG-PCL-Tat-universal negative control siRNAs (siControl) (N/P = 30), and PEG-PCL-Tat-siRelA (N/P = 30) were added separately to BV-2 cells, and RelA mRNA expression after LPS stimulation was evaluated (Figure 3). Treatment with LPS alone, naked siRelA, and PEG-PCL-Tat-siControl showed similar expression levels. PEG-PCL-Tat-siRelA significantly reduced RelA/glyceraldehyde-3-phosphate dehydrogenase (GAPDH) expression. Cells without LPS stimulation also showed low expression levels, comparable to those observed with PEG-PCL-Tat-siRelA.

**Figure 3:**
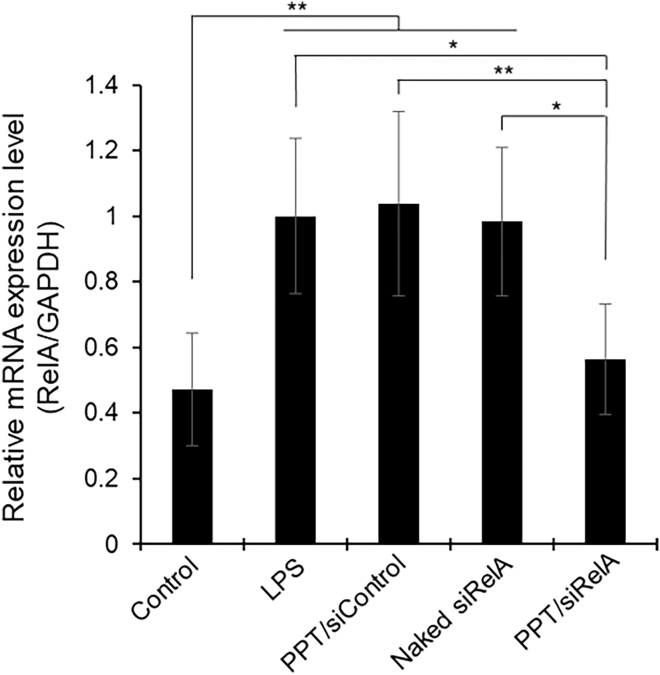
RelA mRNA expression levels in BV-2 cells after treatment with PEG-PCL-Tat-siRelA complexes BV-2 cells were treated with naked siRelA, PEG-PCL-Tat (PPT)-siControl complexes (N/P ratio = 30), or PPT-siRelA complexes (N/P ratio = 30) at a final siRNA concentration of 1.33 μg/mL for 24 h, followed by stimulation with 1 μg/mL lipopolysaccharide (LPS) for 2 h. Total RNA was extracted, and RelA mRNA expression levels were quantified by real-time RT-PCR using SYBR Green. Expression levels were normalized to GAPDH. Data are presented as mean ± SD (*n* = 6). Statistical analysis was performed using one-way ANOVA followed by Tukey’s post hoc test. ∗*p* < 0.05, ∗∗*p* < 0.01.

### Cytotoxicity assessment of PEG-PCL-Tat-siRNA in BV-2 cells and primary cultured hippocampal neurons

The cytotoxicity results of the PEG-PCL-Tat-siControl complex in BV-2 cells are shown in Figures 4A and 4B. All the nanomicelles exhibited survival rates comparable to those of the control, with similar numbers of live and dead cells. In primary cultured hippocampal neurons, lactate dehydrogenase (LDH) release was measured at 4 and 24 h after nanomicelle addition. At 4 h, the LDH absorbance differed significantly between the control and the nanomicelles with an N/P ratio of 30, whereas no significant differences were observed at 24 h. Compared with the tunicamycin stimulation group, which induced approximately 20%–30% cell death, all nanomicelles resulted in lower LDH release (Figures 4C‒4E).

**Figure 4:**
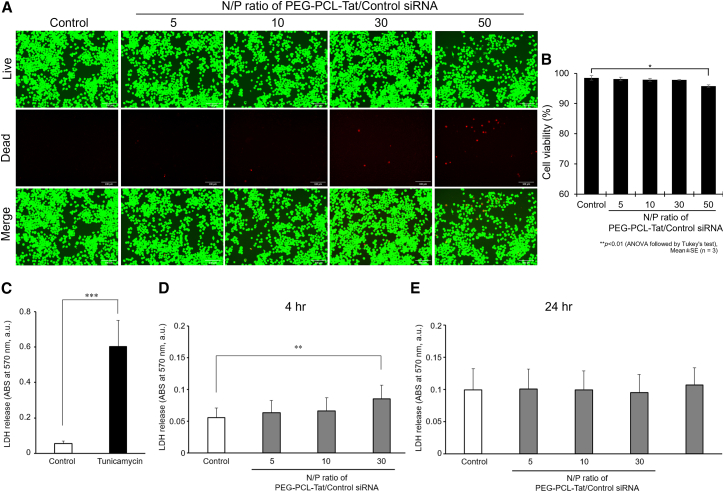
Cytotoxicity of PEG-PCL-Tat-siControl complexes in BV-2 cells and primary cultured hippocampal neurons (A) Representative live/dead fluorescence images of BV-2 cells 24 h after treatment with PEG-PCL-Tat-siControl complexes at the indicated N/P ratios. Live cells were stained with calcein AM (green), and dead cells were stained with ethidium homodimer-1 (red); scale bars, 100 μm. (B) Quantitative analysis of viability in BV-2 cells. Viability was calculated as the percentage of live cells relative to the total number of cells. Data are presented as mean ± SD (*n* = 3). (C–E) LDH release under basal conditions (Control) and after treatment with 1 μg/mL tunicamycin (Tunicamycin) (C), and LDH release after exposure for (D) 4 h and (E) 24 h to PEG-PCL-Tat-siControl complexes at the indicated N/P ratios in primary cultured hippocampal neurons. After incubation with PEG-PCL-Tat-siControl complexes at N/P ratios of 5–30, culture supernatants were collected, and absorbance (ABS) at 570 nm as LDH release was quantified using an LDH-Cytotoxic Test (Wako) according to the manufacturer’s instructions. Tunicamycin was used as a positive control for cytotoxicity because it reduced MTT values by approximately 20%–30% in the preliminary experiments. The absorbance was measured at 570 nm after subtracting the background values. Data are presented as mean ± SD (*n* = 4). Statistical analyses were performed using one-way ANOVA followed by Tukey’s post hoc test in (B), (D), and (E) and by unpaired Student’s *t* test in (C). ∗∗*p* < 0.01, ∗∗∗*p* < 0.0001.

### Brain distribution and quantitative analysis of radiolabeled model siRNA-loaded PEG-PCL-Tat nanomicelles in the ipsilateral and contralateral forebrain after intranasal administration in the MCAO model mice

^14^C-Dextran (Mw: 10,000) was employed as a molecular weight surrogate tracer (model siRNA) of native siRNA to evaluate the quantitative distribution of the PEG-PCL-Tat-model siRNA complex in the ipsilateral (infarcted) and contralateral (normal) forebrain lesions after intranasal administration (Figures S2A‒S2D). The distribution of PEG-PCL-Tat-model siRNA in the infarcted ischemic lesions was significantly higher than that of the naked model siRNA at 15 and 60 min after administration, with the 15-min level being approximately 7-fold higher than that of the naked model siRNA. Although the distribution decreased at 60 min for both groups, the PEG-PCL-Tat-model siRNA complex still showed approximately 3-fold higher levels than the naked model siRNA at this later time point. In contrast, the distribution of the model siRNA in normal lesions showed no difference between the naked model siRNA and the PEG PCL Tat-model siRNA complex. The PEG-PCL-Tat-model siRNA complex tended to exhibit increased distribution levels in the infarcted side (0.67 ID%/g tissue at 15 min and 0.13 ID%/g tissue at 60 min) compared with the normal side (0.48 ID%/g tissue at 15 min and 0.07 ID%/g tissue at 60 min).

### *Ex vivo* fluorescence imaging of Alexa 750-siRNA in the whole brain after intranasal administration of PEG-PCL-Tat-Alexa 750-siRNA in the MCAO model mice

The intracerebral fluorescence distribution 60 min after intranasal administration of the PEG-PCL-Tat-Alexa 750-siRNA complex or naked Alexa 750-siRNA to MCAO mice was examined using the *in vivo* imaging system (Figure 5). In mice treated with naked Alexa 750-siRNA, almost no fluorescent signal was detected in the brain. In contrast, the mice that were administered the PEG-PCL-Tat-Alexa 750-siRNA complex exhibited clear intracerebral accumulation of Alexa 750-siRNA. Notably, fluorescence increased in the left forebrain, including the infarcted region, compared with the normal right forebrain.

**Figure 5:**
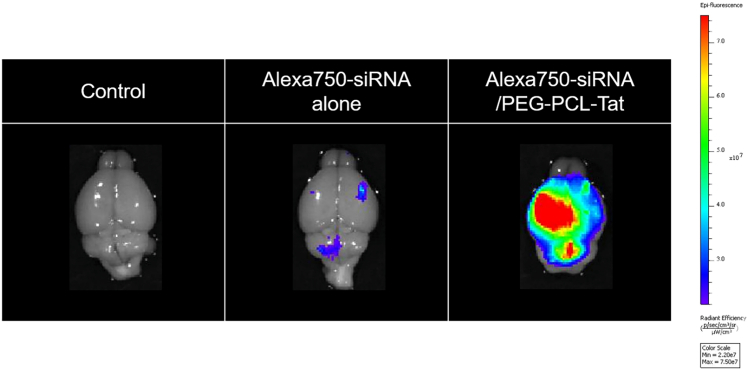
*Ex vivo* fluorescence imaging of Alexa 750-siRNA in MCAO mice after intranasal administration *Ex vivo* fluorescence imaging of Alexa 750-siRNA in MCAO mice 60 min after intranasal administration of naked Alexa 750-siRNA or the PEG-PCL-Tat/Alexa 750-siRNA complex. The fluorescence of Alexa Fluor 750-siRNA in whole brains from the top was observed with the IVIS imaging system. The infarcted hemisphere is shown on the left side of each image.

### Therapeutic effects on transient cerebral ischemia-reperfusion injury in MCAO model mice

The results of the neurological score assessment, observation of 2,3,5-triphenyltetrazolium chloride (TTC)-stained brain slices for infarct localization, and infarct volume calculation following intranasal administration of PEG-PCL-Tat-siRelA (N/P = 30) in MCAO model mice are shown in Figure 6. The group administered with PEG-PCL-Tat-siRelA exhibited significantly improved neurological scores compared with all other groups (Figure 6A). Analysis of TTC-stained serial coronal brain sections revealed extensive infarct lesions in the left hemisphere of the untreated group and the groups treated with PEG-PCL-Tat alone and PEG-PCL-Tat-siControl. Naked siRelA produced a slight reduction in infarct size, whereas PEG-PCL-Tat-siRelA led to a substantial reduction in infarct size (Figure 6B). The infarct volume was calculated from the slice images by using ImageJ, which showed that PEG-PCL-Tat-siRelA administration resulted in a significant reduction compared with the untreated group and the groups treated with PEG-PCL-Tat alone and PEG-PCL-Tat-siControl (Figure 6C).

**Figure 6:**
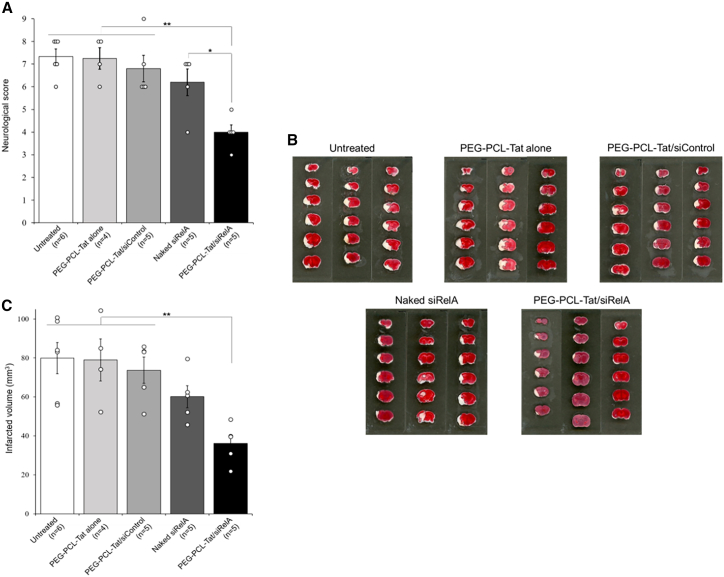
Therapeutic effects of intranasal administration of PEG-PCL-Tat-siRelA in t-MCAO mice (A–C) PEG-PCL-Tat-siRelA or control solutions were administered intranasally 2 h after reperfusion. Neurological score assessment and infarct volume analysis were performed 22 h after occlusion. (A) Neurofunctional scores (0–9). (B) Representative TTC-stained coronal brain sections. (C) Quantitative analysis of infarct volume calculated from six consecutive 1-mm-thick coronal sections. Data are presented as mean ± SD, with individual data points overlaid on the bar graphs (*n*, as indicated in each graph). Statistical analysis was performed using a one-way ANOVA followed by Tukey’s post-hoc test. ∗*p* < 0.05, ∗∗*p* < 0.01.

### The mRNA expression levels of RelA and inflammatory cytokines in the brain tissue of MCAO model mice

The sequence-specific effects of PEG-PCL-Tat-siRelA (N/P = 30) on RelA expression were evaluated based on the mRNA expression levels of RelA and other inflammatory cytokines (Figure 7). Quantitative reverse-transcription PCR (RT-qPCR) performed 22 h after reperfusion in MCAO mice subjected to 2 h of ischemia revealed that the relative mRNA expression levels of RelA (Figure 7A), TNF-α (Figure 7B), IL-1β (Figure 7C), and IL-6 (Figure 7D), normalized to GAPDH, increased by 2- to 15-fold compared with non-MCAO controls. Intranasal administration of PEG-PCL-Tat-siRelA reduced these mRNA levels to 20%–50% of untreated levels. PEG-PCL-Tat-siControl and naked siRelA had no significant effects, whereas significant reductions in all inflammatory cytokines compared with the untreated condition were observed only with PEG-PCL-Tat-siRelA administration and in the non-MCAO control.

**Figure 7:**
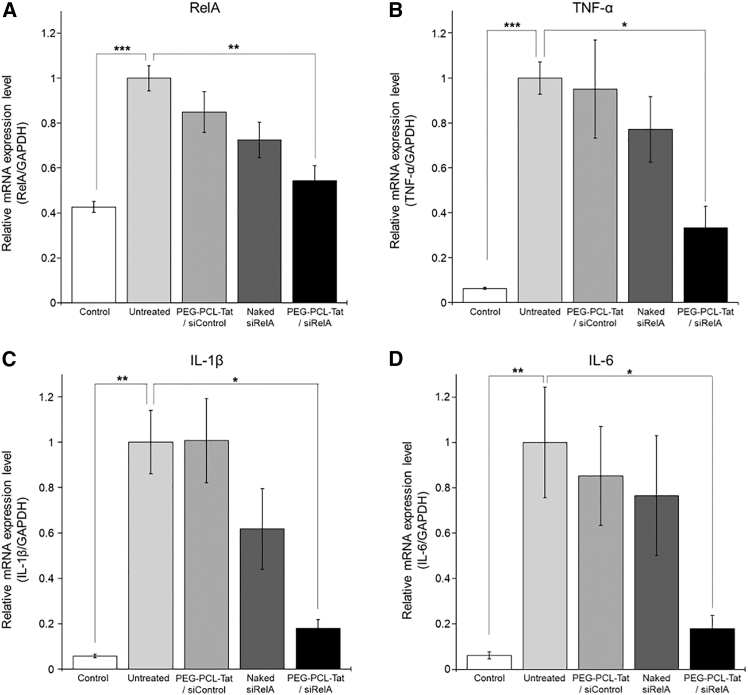
mRNA expression levels of inflammatory cytokines in the ischemic hemisphere of t-MCAO mice (A–D) At 22 h after reperfusion, the left cerebral hemisphere was collected, and relative mRNA expression levels were determined by RT-qPCR using the ΔΔCt method, with GAPDH as the endogenous control and normalization to the untreated group. (A) RelA mRNA expression. (B) TNF-α mRNA expression. (C) IL-1β mRNA expression. (D) IL-6 mRNA expression. Data are presented as mean ± SE (*n* = 8). Statistical analysis was performed using one-way analysis of variance (ANOVA), followed by Dunnett’s post hoc test versus the untreated group. ∗*p* < 0.05, ∗∗*p* < 0.01, ∗∗∗*p* < 0.001.

## Discussion

This study demonstrated that the intranasal administration of PEG-PCL-Tat nanocarriers loaded with siRelA enhanced brain delivery, thereby reducing infarct volume and suppressing cytokine production in MCAO mice. Cerebral infarction progresses through neuroinflammation driven by microglial activation.^4^^,^^5^ NF-κB is a key regulator of the expression of pro-inflammatory cytokines, including TNF-α, IL-1β, and IL-6, and is critically involved in the exacerbation of cerebral infarction.^35^ NF-κB functions as a dimer and activates the transcription of inflammatory cytokine-responsive genes upon nuclear translocation,^36^ with RelA-mediated signaling being particularly important for inflammatory responses. siRNA-mediated gene silencing has been explored as a therapeutic strategy for inflammatory diseases, and we have previously reported the efficacy of RelA-targeted siRNA combined with functional peptides in a mouse model of atopic dermatitis.^37^^,^^38^ However, the effective delivery of siRNA to the brain remains a major challenge because of the BBB. Accordingly, this study focused on intranasal administration of PEG-PCL-Tat nanocarriers with enhanced brain-delivery properties to investigate the therapeutic potential and knockdown efficiency of siRelA in an acute cerebral ischemia-reperfusion model.

The PEG-PCL-Tat-siRelA complex exhibited a decrease in particle size and an increase in zeta potential as the N/P ratio increased (Table 1). These results are consistent with those of previous studies that varied the polymer/plasmid DNA ratio.^39^ The increase in zeta potential was due to the higher proportion of the Tat peptide, which contains arginine and lysine residues rich in positive charges. The stronger positive charge also enhances electrostatic interactions with negatively charged siRelA, leading to tighter condensation of the complex.^40^^,^^41^ Simultaneously, a higher surface potential improves the dispersion stability, preventing excessive aggregation. Evaluation of CSF stability of the complex prepared at an N/P ratio of 30 revealed minimal degradation over 24 h (Figure 1), indicating that complexation with PEG-PCL-Tat enhanced siRelA stability. These micelles are generally reported to exhibit extremely low critical micelle concentrations (CMCs), typically on the order of approximately 10^−7^ M^42^ and are, therefore, regarded as stable against dilution. In addition, despite the presence of positive surface charge derived from Tat, colloidal stability is maintained due to steric stabilization provided by PEG chains. However, because the stability assay was based on SYBR Green staining, it does not directly assess the sequence integrity of siRNAs. Nevertheless, the PEG-PCL-Tat-siRelA complex exhibited biological activity *in vivo*, suggesting that at least a fraction of the siRNA retained its sequence integrity. Confirmation of siRNA integrity is important for further improving the delivery efficiency and therapeutic efficacy in future studies.

In a cellular uptake experiment using BV-2 cells, naked FAM-siRNA showed minimal cellular uptake, whereas PEG-PCL-Tat-FAM-siRNA complexes exhibited marked uptake at all N/P ratios (Figures 2 and S1). FAM-siRNA uptake increased in an N/P ratio-dependent manner. The enhanced uptake at higher N/P ratios may be attributed to the increased cationic surface charge, which likely strengthens electrostatic interactions with the negatively charged BV-2 cell membrane, thereby promoting engagement with cell surface proteoglycans and facilitating subsequent endocytic processes. BV-2 cells were stimulated with LPS, and the mRNA expression level of RelA, normalized to that of the housekeeping gene GAPDH, was quantified. A significant reduction in RelA mRNA expression was observed in the PEG-PCL-Tat-siRelA-treated group (Figure 3). LPS stimulation has been reported to upregulate RelA expression in BV-2 cells.^43^^,^^44^ Consistent with these reports, RelA mRNA levels were elevated in the LPS-alone, PEG-PCL-Tat-siControl, and naked siRelA groups compared with the untreated control group. In contrast, treatment with PEG-PCL-Tat-siRelA significantly suppressed RelA mRNA expression, suggesting the effective intracellular delivery of siRelA and subsequent gene silencing.

No sustained cytotoxic effects were observed in BV-2 cells or primary cultured hippocampal neurons, even at an N/P ratio of 30 for PEG-PCL-Tat-siRNA (Figure 4). Although PEG-PCL-Tat-siControl complexes at an N/P ratio of 30 caused a slight but significant increase in LDH release at 4 h in primary cultured hippocampal neurons, this increase was not observed at 24 h. This transient response may reflect temporary membrane perturbation or an early stress response associated with the positively charged nanomicelle complex, rather than sustained cytotoxicity. Nevertheless, further safety evaluation using additional cytotoxicity markers and longer or repeated exposure condition is necessary. Although cationic surface charge is widely recognized as a critical determinant of nanoparticle-associated cytotoxicity,^45^^,^^46^ previous studies have reported no detectable cytotoxicity of nanoparticles with positive zeta potentials ranging from +10 to +40 mV.^47^^,^^48^^,^^49^ Given that the zeta potential of the present formulation was approximately +20 mV, these findings are consistent with those of previous reports. While the surface charge contributes to cytotoxic potential, cellular responses are influenced by multiple physicochemical parameters, including particle composition and size, suggesting that a moderately positive surface charge may be tolerated depending on the formulation characteristics. Evaluation of *in vivo* cytotoxicity is an important issue for clinical translation, and further detailed studies, including histopathological analyses, are required in future investigations.

In the evaluation of brain distribution following intranasal administration in the MCAO mouse model, the PEG-PCL-Tat-model siRNA-treated group showed significantly higher levels in the ischemic brain region at 15 and 60 min than the naked model siRNA group (Figures S2A and S2C). In our previous study, we demonstrated that intranasally administered siRNA complexed with nanomicelles composed of PEG-PCL and an arginine-rich peptide could reach the brain after crossing the nasal epithelium via the olfactory and trigeminal nerve pathways.^50^ A similar delivery mechanism likely applies to the PEG-PCL-Tat used in the present study, indicating that intranasal administration enables the delivery of model siRNA to the brain, even under ischemia-reperfusion conditions. In the normal regions, no significant difference in the model siRNA distribution was observed between the naked model siRNA and the PEG-PCL-Tat-model siRNA complex (Figures S2B and S2D). Local pharmacokinetics in ischemic tissues are known to be altered by BBB disruption, neuroinflammation, and increased vascular permeability.^51^ Therefore, the high distribution to the ischemic hemisphere observed in this study may have been influenced by these pathophysiological changes. However, naked model siRNA alone showed no difference in the distribution between the normal and infarcted regions, whereas PEG-PCL-Tat-model siRNA complex showed approximately 2-fold higher distribution level of model siRNA in the infarcted region (0.13 ID%/g tissue) than that in the normal region (0.07 ID%/g tissue) at 60 min after administration (Figures S2C and S2D). These findings suggest that PEG-PCL-Tat not only enables nose-to-brain delivery of siRNAs but may also enhance the distribution and retention of siRNAs within the infarcted lesion. Although ^14^C-dextran (Mw, 10,000) was used as a molecular weight surrogate tracer for model siRNA, and its behavior may not fully replace that of native siRNAs, we previously reported that PEG-PCL-Tat complexes loaded with dextran and siRNA exhibited comparable particle size (approximately 50–80 nm) and positively charged zeta potential (approximately +10 to –20 mV),^34^ consistent with those observed in the present study. Nevertheless, differences in molecular conformation and charge characteristics between dextran and siRNA should be considered when interpreting the quantitative biodistribution results. The absolute delivery efficiency of native siRNA cannot be determined solely from the ^14^C-dextran distribution data. To further validate these findings, *ex vivo* fluorescence imaging was performed, which corroborated them by showing clear intracerebral accumulation of Alexa 750-siRNA when delivered via PEG-PCL-Tat, with stronger fluorescence in the left forebrain, including the infarcted region, than in the normal right forebrain (Figure 5). These findings suggest that, in addition to the pathophysiological changes associated with cerebral ischemia, enhanced membrane permeability conferred by PEG-PCL-Tat may also contribute to the increased distribution. An effect analogous to the electron paramagnetic resonance (EPR) effect of nanoparticles may also be involved, although the detailed mechanism remains unclear and requires further investigation.

Neurological evaluation of ischemia-reperfusion injury in the MCAO mouse model showed that intranasal administration of PEG-PCL-Tat-siRelA significantly improved the neurological scores compared with the other groups (Figure 6A). Quantitative analysis of infarct volume using ImageJ did not show a statistically significant difference compared with the naked siRelA group. However, image-based assessment showed the greatest reduction in infarct volume in the PEG-PCL-Tat-siRelA-treated group compared with the other groups (Figures 6B and 6C). Following cerebral ischemia, activation of the NF-κB pathway rapidly promotes cell death and edema formation, and RelA is one of the most prominently activated components in the MCAO model.^52^^,^^53^ In this study, the combination of PEG-PCL-Tat and intranasal administration enabled the delivery of siRelA to the ischemic brain region, which was associated with the suppression of RelA-mediated inflammatory signaling.

Finally, the knockdown effects on the mRNA expression of RelA and inflammatory mediators (TNF-α, IL-1β, and IL-6) were evaluated in the MCAO mouse model. Using Dunnett’s test with the untreated group as the control, significant reductions in mRNA expression were observed only in the PEG-PCL-Tat-siRelA intranasal administration group and in the non-ischemic untreated group for all four factors (Figures 7A–7D). A limitation of this study is that the analysis was limited to the gene expression level, and reductions in inflammatory mediators at the protein level were not evaluated. However, suppression of RelA and downstream inflammatory cytokine mRNA expression, together with improvements in infarct volume and neurological outcomes, were observed. In addition, previous studies have reported reductions in inflammatory mediator expression following RelA mRNA suppression.^54^ Collectively, these findings suggest that PEG-PCL-Tat micelles enabled mRNA knockdown in the ischemic region and associated functional improvement.

This study demonstrated the potential utility of PEG-PCL-Tat micelles as nose-to-brain siRNA therapeutic nanocarriers for ischemia-reperfusion injury. The nose-to-brain route has been proposed to involve both the olfactory and trigeminal pathways. Because rodents possess a larger olfactory region in the nasal cavity than humans,^25^^,^^55^^,^^56^ its efficacy observed in the present mouse study may have been overestimated. However, several recent studies have reported its successful application in non-human primates,^57^^,^^58^ and drug transport via both the olfactory and trigeminal pathways have been observed. Although whether efficacy comparable to that observed in rodents can be achieved in primates remains unclear, these findings suggest that nose-to-brain drug delivery may also be feasible in primates. Further studies are required to elucidate the underlying mechanisms and improve the efficacy of this delivery strategy. Recent studies have reported enhanced brain delivery through increased adhesion to the nasal mucosa^59^^,^^60^ and improved brain targeting via ligand modifications.^61^^,^^62^ Particle size is also a critical consideration for intranasal administration. For example, although ultrasmall nanoparticles of approximately 15 nm are advantageous because they can traverse the paracellular space of the olfactory epithelium, their small size makes preparation technically challenging, their *in vivo* behavior remains unclear, and their application in drug delivery is considered limited.^63^ Although the total amount of drug reaching the brain was low, measurable therapeutic effects were achieved in the present study. Previous reports have shown that reductions in infarct volume and improvements in neurological outcomes are more pronounced when evaluated several days after treatment than at 24 h,^64^^,^^65^^,^^66^ suggesting that repeated administration or longer observation periods may yield enhanced therapeutic efficacy. In addition, long-term formulation stability will be an important consideration for future clinical translation. Further investigation is required to consider mucosal adhesion, surface modification, particle size control, and evaluation strategies.

### Conclusion

In this study, intranasal administration of siRNA-loaded PEG-PCL-Tat complexes to MCAO mice enabled efficient delivery to the ischemic brain regions, resulting in reduced infarct volume and effective knockdown of inflammation-related molecules. *In vitro* experiments with BV-2 cells demonstrated efficient cellular uptake of PEG-PCL-Tat, with no apparent cytotoxicity. Intranasal administration is a non-invasive delivery route that bypasses the BBB and enables simple, repeated dosing to target the central nervous system. Therefore, the combination of PEG-PCL-Tat and intranasal administration may facilitate effective drug delivery for refractory brain disorders, such as cerebral ischemia-reperfusion injury, and represents a promising therapeutic strategy for cerebrovascular diseases in the future.

## Materials and methods

### Materials and cells

The anti-RelA siRNA (siRelA; sense: 5′-GGU GCA GAA AGA AGA CAU UdTdT-3′, antisense: 5′-AAU GUC UUC UUU CUG CAC CdTdT-3′) and FAM-labeled siRNA (FAM-siRNA; sense: 5′-6FAM-AUC CGC GCG AUA GUA CGU AdTdT-3′, antisense: 5′-UAC GUA CUA UCG CGC GGA UdTdT-3′) were purchased from CosmoBio Co., Ltd. (Tokyo, Japan). Alexa 750-modified siRNA (Alexa 750-siRNA; sense: 5′-Alexa750-UUC UCC GAA CGU GUC ACG U dTdT-3′, antisense: 5′-ACG UGA CAC GUU CGG AGA UdTdT-3′) was purchased from Thermo Fisher Scientific Inc. (MA, USA). siRelA targeted 5′-AAG GTG CAG AAA GAA GAC ATT-3′ in the RelA sequence of the mice. Universal negative control siRNAs (siControl; sense: 5′-AUC CGC GCG AUA GUA CGU AdTdT-3′, antisense: 5′-UAC GUA CUA UCG CGC GGA UdTdT-3′) were obtained from Nippon Gene (Tokyo, Japan). PEG-PCL-Tat was synthesized using methoxy poly (ethylene glycol)-block-poly (ε-caprolactone) (2k-2k) (PEG-PCL block co-polymers) (Sigma-Aldrich Co., Milwaukee, WI, USA) and Tat peptide (Gly-Arg-Lys-Lys-Arg-Arg-Gln-Arg-Arg-Arg-Gly) (BEX Co., Ltd., Tokyo, Japan). Dextran (carboxyl-^14^C) (^14^C -dextran, molecular weight: 10,000, 3.6 mCi/g; purity >99%) as model siRNA was purchased from American Radiolabeled Chemicals Inc. (St Louis, MO, USA). All other chemical solvents and reagents were of commercial grade and were used without any further purification. BV-2 cell line was provided by Professor Eui-Ju Choi of the Department of Life Science, Korea University, Seoul, Republic of Korea. The cells were cultured in high-glucose Dulbecco’s modified Eagle’s medium (DMEM; Gibco, California, USA) supplemented with 10% fetal bovine serum (FBS) at 37°C in a humidified incubator with 5% CO_2_.

### Primary culture of hippocampal neurons

All animal experiments (experiment number #AP24PHA013-1) were performed in accordance with the Guidelines for Animal Experiments at Nihon University. Cultured mouse hippocampal neurons were prepared as previously described.^67^ Briefly, the hippocampi were isolated from the brains of embryonic day 18 (E18) CB57BL/6J mice and treated with neuronal dissociation solutions (FUJIFILM Wako Pure Chemical Corporation). The cells were plated at a density of 5.0 × 10^5^ cells/cm^2^ on poly-L-lysine-coated 8-well culture glass (AGC Techno Glass Co., Ltd.) and a 3.5-cm culture dish (Corning) and maintained for 7 days in neurobasal medium (Gibco) containing 0.5 mM L-glutamine and 2% B27 supplement (Gibco).

### Animals

All animal experiments (experiment numbers #AP18PHA013-3 and AP20PHA005-1) were carried out according to the guidelines approved by the Nihon University Animal Care and Use Committee (Tokyo, Japan). Four-week-old male mice (of the Deutschland, Denken, and Yoken [ddY] strains) were obtained from Japan SLC, Inc. (Shizuoka, Japan). The mice were provided water *ad libitum* under controlled temperature/humidity conditions. Mice weighing 25–35 g were used for the experiments after being pre-fed for at least 3 days.

### Synthesis of Tat peptide conjugated to PEG-PCL

PEG-PCL-Tat was synthesized, and the conjugation reaction was confirmed, as previously described.^32^ Briefly, the Tat peptide and the PEG-PCL block co-polymer were dissolved in dimethylformamide. Water-soluble carbodiimide hydrochloride (WSCI) and 4-dimethylaminopyridine were added, and the mixture was incubated at room temperature for 24 h. The terminal –OH group on the PEG-PCL block co-polymer and the Gly COOH group on the C terminus of the Tat peptide ester bond formed an ester bond. The reaction solution was transferred to a dialysis membrane suitable for use with organic solvents (Spectra/Por Dialysis Membranes, molecular weight cut‑off: 3,500) and dialyzed against distilled water for 24 h. The product was lyophilized to yield PEG-PCL-Tat.

### Preparation of PEG-PCL-Tat-siRelA complexes

The siRelA was dissolved in nuclease-free water to prepare a stock solution. PEG-PCL-Tat was dissolved in HEPES buffer at concentrations required to achieve the designated N/P ratios. Equal volumes of the siRelA solution were gently added dropwise to the PEG-PCL-tat solution, then mixed with a vortex mixer. The resulting mixture was allowed to stand at room temperature (25°C) for 30 min, yielding PEG-PCL-Tat/siRelA solutions with N/P ratios of 5, 10, 20, or 30. Naked siRelA was prepared by mixing siRelA solution with an equal volume of HEPES buffer. Separately, siControl was dissolved in nuclease-free water and mixed with an equal volume of the PEG-PCL-Tat solution to obtain a PEG-PCL-Tat-siControl solution (N/P ratios, 5–30). All samples were prepared at a final siRNA concentration of 400 μg/mL. The mean particle size and zeta potential of the PEG-PCL-Tat-siRelA complexes with N/P ratios of 5–30 were measured using a NICOMP 380ZLS (Particle Sizing Systems Co., Santa Barbara, CA, USA).

### Stability of PEG-PCL-Tat-siRelA complexes in mouse CSF

Nuclease-free water was added to 2.5 μL of naked siRelA or PEG-PCL-Tat-siRelA solution to obtain a final volume of 1 mL. A 100 μL aliquot from each solution was collected as the 0 h sample. Subsequently, 10 μL of CSF collected from mice was added to the remaining 900 μL of each sample solution. Aliquots of 100 μL were collected after 0.5, 1, 2, 4, 6, 12, and 24 h. A 0.05 mM dextran sulfate solution (50 μL) was added to each aliquot, followed by incubation for 15 min. Each 100 μL aliquot was transferred to a 96-well plate, and 50 μL of SYBR Green solution diluted 1,000-fold with phosphate-buffered saline (PBS) was added. After incubation for 15 min, fluorescence was measured using a microplate reader (FLUOstar Omega, BMG LABTECH, Germany) with excitation at 494 nm and emission at 521 nm.

### Intracellular uptake of PEG-PCL-Tat-siRNA in BV-2 cells

BV-2 cells (1 × 10^4^ cells/well) were maintained in Dulbecco’s modified Eagle’s medium (DMEM) supplemented with 10% FBS at 37°C in a humidified incubator with 5% CO_2_. The cells were seeded onto 8-well chamber slides (Thermo Fisher Scientific). After 24 h, the culture medium was removed, and the cells were treated with naked FAM-siRNA or PEG-PCL-Tat-FAM-siRNA complexes at N/P ratios of 5, 10, and 30. The siRNA concentration in the culture medium was adjusted to 1.33 μg/well for all samples. After 4 h of incubation, the cells were washed thrice with Hank’s balanced salt solution (HBSS). The medium was replaced with DMEM containing 10% FBS, followed by the addition of Hoechst 33258 and incubation for 30 min. The cells were washed thrice with HBSS, mounted using the mounting medium, and observed under a fluorescence microscope (BZ-X810; Keyence, Tokyo, Japan).

### *In vitro* mRNA expression analysis in BV-2 cells

BV-2 cells were seeded onto 6-cm culture dishes and incubated in DMEM supplemented with 10% FBS. The following day, naked siRelA, PEG-PCL-Tat-siControl (N/P ratio = 30), or PEG-PCL-Tat-siRelA (N/P ratio = 30) were added to the culture medium at a concentration of 1.33 μg/mL. After 24 h of incubation, the cells were stimulated with 1 μg/mL LPS-containing DMEM for 2 h. The cells were then collected, and total RNA was extracted using a High Pure RNA Isolation Kit (Nippon Genetics, Tokyo, Japan) according to the manufacturer’s instructions. Complementary DNA (cDNA) was synthesized from 1.0 μg of total RNA by using reverse transcriptase (Wako, Osaka, Japan). Quantitative real-time PCR was performed on a Stratagene MX3000 real-time PCR system, using SYBR Green PCR Reagent (Promega, Madison, WI, USA). The primer sequences were as follows: RelA (forward), 5′-GCGTACACATTCTGGGGAGT-3′, and RelA (reverse), 5′-ACCGAAGCAGGAGCTATCAA-3′; GAPDH (forward), 5′-TCAACAGCAACTCCCACTCTTCCA-3′, and GAPDH (reverse), 5′-ACCCTGTTGCTGTAGCCGTATTCA-3′.

### Cytotoxicity assay in BV-2 cells and primary cultured hippocampal neurons

BV-2 cells (1 × 10^4^ cells/well) were seeded onto 8-well chamber slides (Thermo Fisher Scientific). The following day, the medium was removed, and the cells were transfected with PEG-PCL-Tat-siControl at N/P ratios of 5–30. After 24 h, the medium containing PEG-PCL-Tat-siControl was removed, and the cells were washed three times with HBSS. After washing, DMEM containing 10% FBS was added. Live and dead cells were stained with 2 μM calcein AM and 4 μM ethidium homodimer-1 according to the manufacturer’s protocol (#L3224, Thermo Fisher Scientific, MA, USA). Fluorescence images were obtained using an all-in-one fluorescence microscope (BZ-X810; KEYENCE, Osaka, Japan). Live and dead cells were counted, and cell viability was calculated.

The cytotoxicity range was evaluated using LDH released into the medium during exposure to nanomicelles. Tunicamycin (1 μg/mL; Sigma-Aldrich, MO, USA) was used as a positive control for cytotoxicity. After cultured mouse hippocampal neurons were incubated with nanomicelles or tunicamycin for 4 h, they were incubated for an additional 20 h in culture medium under a humidified atmosphere containing 5% CO_2_ at 37°C. The supernatants were collected in new plates (AGC Techno Glass Co., Ltd.), and LDH release was determined using an LDH-Cytotoxic Test Wako kit (FUJIFILM Wako, Pure Chemical Corporation), as described previously.^68^ Absorbance was measured at 570 nm by using a microplate reader (SH-1000Lab, Corona Electric). The background absorbance of the culture medium was subtracted from each sample’s absorbance.

### Creating a transient MCAO model in mice by filamentous occlusion

Focal cerebral ischemia was induced by intraluminal suture occlusion of the middle cerebral artery, as previously described,^69^ with some minor modifications. Briefly, the mice were placed under anesthesia (initiation: 4%, maintenance: 2%) in the supine position, and the nasal region was covered with an openable inhalation mask (SN-487-70-09, Shinano Seisakusho, Tokyo, Japan). The left common carotid artery (CCA), the external carotid artery (ECA), and the internal carotid artery (ICA) were exposed through a midline incision under a microscope. The occipital and superior thyroid arteries were electrocoagulated. The CCA and the ICA were clamped using microvascular clips. A 6–0 monofilament nylon suture was inserted retrogradely into the ICA via arteriectomy of the left ECA. The suture was gently advanced into the ICA, 9 mm from the bifurcation of the CCA, to block the origin of the MCA. After 2 h of occlusion, the intraluminal suture was carefully withdrawn to allow reperfusion, and the cerebral blood flow was recovered.

### Intranasal administration procedure in mice

The mice were placed under isoflurane anesthesia (initiation, 4%; maintenance, 2%) in the supine position, and the nasal region was covered with an openable inhalation mask (SN-487-70-09, Shinano Seisakusho, Tokyo, Japan). After opening the mask’s silicone cap, each mouse was administered 25 μL of the sample in 2 μL doses at 1-min intervals, alternating between the left and right nostrils, with the final administration volume adjusted to 1 μL. Droplets from the micropipette tip were placed near the nasal cavity, allowing the mice to inhale and absorb the solution naturally.

### Evaluation of brain distribution following intranasal administration of radiolabeled model siRNA-PEG-PCL-Tat in MCAO model mice

^14^C-Dextran (Mw: 10,000) was used as a model for siRNA. A ^14^C-dextran solution was prepared by diluting the ^14^C‑dextran with water before mixing with PEG‑PCL‑Tat. The PEG-PCL-Tat was gently added dropwise to the ^14^C-dextran solution, and the two were mixed in equal volumes. The mixture was allowed to stand at room temperature for 30 min to obtain the PEG-PCL-Tat-model siRNA. The concentration of ^14^C-dextran was adjusted to 2 μCi/mL, and the molar ratio of amino groups of PEG-PCL-Tat to carboxyl groups of the model siRNA was set to 1:5. A model siRNA solution was prepared by adding an equal volume of HEPES buffer to the ^14^C-dextran solution.

After establishment of the MCAO model, the mice were reperfused, and 25 μL of the prepared PEG-PCL-Tat-model siRNA or model siRNA solution was administered intranasally 2 h after occlusion. Brains were harvested 15 and 60 min after intranasal administration, and the ipsilateral (infarcted side) and contralateral (non-treated side) forebrains were collected. After weighing, brain samples were solubilized in 1 mL of Solusol (National Diagnostics, GA, USA) at 55°C for 1–2 h, then 10 mL of Hionic-fluor (Waltham, MA, USA) was added. The radioactivity of ^14^C-dextran in the brain was measured using a liquid scintillation counter (Tri-Carb 4810TR, PerkinElmer, MA, USA). To calculate the percentage of ^14^C-dextran in the ipsilateral or the contralateral forebrain relative to the administered dose (%ID/g brain), the radioactivity of ^14^C-dextran per gram of brain tissue was divided by the radioactivity of ^14^C-dextran in the administered solution.

### *Ex vivo* imaging in the whole brain following intranasal administration of Alexa 750-siRNA-PEG-PCL-Tat in MCAO model mice

After the establishment of the MCAO model mice, 25 μL of the prepared PEG-PCL-Tat/Alexa 750-siRNA or naked Alexa 750-siRNA (equivalent to 10 μg of Alexa-siRNA) was administered intranasally 2 h after occlusion. Sixty minutes after administration, the mice were anesthetized and transcardially perfused with saline to remove blood from the cerebral vasculature, after which the brains were collected. The fluorescence of Alexa 750-siRNA in the whole brain was observed using an IVIS imaging system (PerkinElmer, Inc., Waltham, MA, USA) with an excitation/emission filter of 740/790 nm (setting value; binning: 2, field of view: D, F/stop: 2).

### Evaluation of therapeutic effects by intranasal administration of PEG-PCL-Tat-siRelA in t-MCAO mice

After MCAO induction, 25 μL of each prepared sample was administered intranasally 2 h after occlusion. Neurofunctional evaluation was performed 22 h after reperfusion, followed by brain tissue collection. Neurofunctional evaluation was conducted to assess the severity of ischemia-reperfusion injury, as described in previous studies.^70^ The evaluation consisted of four parameters: spontaneous activity, left drifting during displacement, parachute reflex, and resistance to left forepaw stretching, with a total score ranging from 0 to 9.

The excised brains were cut coronally into 1-mm-thick sections from the anterior end, using a brain slicer. To measure the infarct area, coronal brain slices were stained by immersion in a 2% TTC (Sigma-Aldrich, MO, USA) solution at 37°C for 15 min. The stained serial coronal brain sections were analyzed using ImageJ software. Infarct volume (mm^3^) was calculated from the infarct areas of six consecutive coronal brain sections, excluding the olfactory bulb.

### Evaluation of mRNA expression levels of RelA and inflammatory cytokines in the brain tissue of t-MCAO model mice

At 22 h after reperfusion, the left cerebral hemisphere (infarcted ischemic side) was excised from the mice. Total RNA was extracted by homogenizing the tissue with RNAiso Plus (Takara Bio Inc., Otsu, Shiga, Japan), using a Power Masher II (Nippi Inc., Tokyo, Japan). After DNase treatment (Nippon Genetics Co., Ltd., Tokyo, Japan), 1 μg of purified total RNA was used as a template for cDNA synthesis using ReverTra Ace (Toyobo Co., Ltd., Osaka, Japan). Reverse transcription was performed using an iCycler Thermal Cycler (Bio-Rad Laboratories, Hercules, CA, USA) at 42°C for 40 s, followed by 99°C for 5 s.

PCR (RT-qPCR) was performed using the Stratagene MX3000 Real-Time PCR System (Agilent Technologies, Santa Clara, CA, USA). Each reaction mixture contained 2 μL of synthesized cDNA, gene-specific primers, SYBR Green PCR reagent (Promega, Madison, WI, USA), and nuclease-free water. The thermal cycling conditions were as follows: initial denaturation at 95°C for 30 s, followed by 40 cycles of denaturation at 95°C for 10 s and annealing at 58°C for 10 s. Subsequently, the dissociation was performed at 72°C for 20 s and 95°C for 15 s.

Cycle threshold (Ct) values were used to calculate relative mRNA expression levels according to the ΔΔCt method. Ct values were normalized to the endogenous control gene GAPDH. ΔΔCt values were calculated by subtracting the ΔCt of the calibrator sample (control group) from that of each experimental sample, and relative expression levels of target genes were estimated compared with the untreated group.^71^ The primer sequences were as follows: RelA (forward), 5′-GCGTACACATTCTGGGGAGT-3′; RelA (reverse), 5′-ACCGAAGCAGGAGCTATCAA-3′; TNF-α (forward), 5′-GTCGTAGCAAACCACCAAGTGG-3′; TNF-α (reverse), 5′-GAGATAGCAAATCGGCTGACGG-3′; IL-1β (forward), 5′-GCCACCTTTTGACAGTGATGA-3′; IL-1β (reverse), 5′-TGTTGATGTGCTGCTGCGAGA-3′; IL-6 (forward), 5′-TCCTCTCTGCAAGAGACTTCCATCC-3′; IL-6 (reverse), 5′-AAGCCTCCGACTTGTGAAGTGGT-3′; GAPDH (forward), 5′-TCAACAGCAACTCCCACTCTTCCA-3′; and GAPDH (reverse), 5′-ACCCTGTTGCTGTAGCCGTATTCA-3′.

### Statistical analysis

All statistical analyses were performed using EZR,^72^ a graphical user interface for R, and a modified version of the R Commander designed to add statistical functions frequently used in biostatistics. Student’s *t* test was used for comparisons between two groups. For multiple group comparisons, analysis of variance (ANOVA) followed by Dunnett’s or Tukey’s tests were applied, as appropriate. Statistical significance was set at *p* < 0.05.

## Data and code availability

The data that support the findings of this study are available from the corresponding author upon reasonable request. All relevant data are included within the article and its supplemental information files.

## Acknowledgments

We are grateful to Dr. Hisako Ibaraki of the Tokyo University of Pharmacy and Life Sciences for *ex vivo* imaging; Dr. Shingo Iioka, Mr. Yuta Hidaka, Mr. Keigo Uezu, Ms. Ryo Asayama, and Mr. Masahiro Yoshino of 10.13039/100007683Nihon University for their technical assistance; and 10.13039/501100001691JSPS KAKENHI (24K22407, 23K28433, 20H04537) and Nagai Memorial Research Scholarship from the Pharmaceutical Society of Japan for a doctoral scholarship.

## Author contributions

Conceptualization, T.K.; data curation, M.F., T.O., Y.K., and T.K.; methodology, M.F., T.O., T.N., Y.K., and T.K.; investigation, M.F., T.O., T.N., Y.K., and T.K.; validation, M.F., T.O., T.N., Y.K., and T.K.; visualization, M.F., T.O., Y.K., and T.K.; writing – original draft, M.F. and T.O.; writing – review & editing, T.N., T.Y., Y.K., T.S., and T.K.; supervision, Y.K., T.S., and T.K.; project administration, T.K.

## Declaration of interests

The authors declare no conflicts of interest.

## Supplemental information

Document S1. Figures S1 and S2

Document S2. Article plus supplemental information
