# Supplementary material for: Intranasal Tat-modified PEG-PCL nanomicelles delivering anti-RelA siRNA attenuate ischemia-reperfusion injury
Source: Mol Ther Nucleic Acids. 2026 Jul 21;37(3):103028. doi: 10.1016/j.omtn.2026.103028 (PMC13475413; doi:10.1016/j.omtn.2026.103028)
Supplement: Supplementary file 1 [file mmc1.pdf]

OMTN, Volume *37*

Supplemental information

Intranasal Tat-modified PEG-PCL nanomicelles delivering anti-RelA siRNA attenuate ischemia-reperfusion injury

Mitsuyoshi Fukuda, Takayuki Oguma, Taiki Nagatomo, Taiki Yamaguchi, Yasuhiro Kosuge, Toyofumi Suzuki, and Takanori Kanazawa

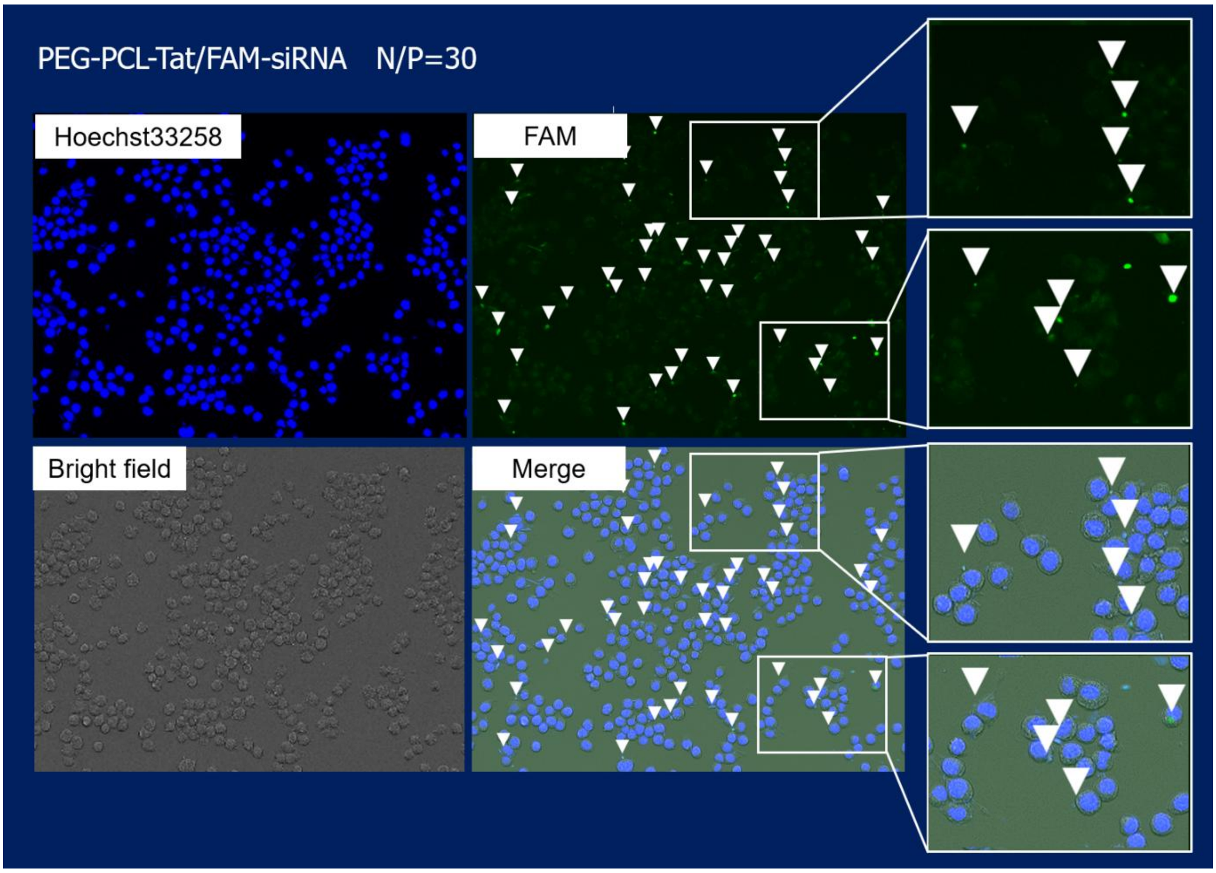

Figure S1. Representative enlarged fluorescence microscopy images of BV-2 cells incubated with PEG-PCL-Tat/FAM-siRNA complexes at N/P = 30 for 4 h. Green fluorescence indicates FAM-siRNA, and nuclei were counterstained with Hoechst 33258 (blue). White arrowheads indicate representative intracellular FAM-siRNA signals. Enlarged views of selected regions are shown on the right.

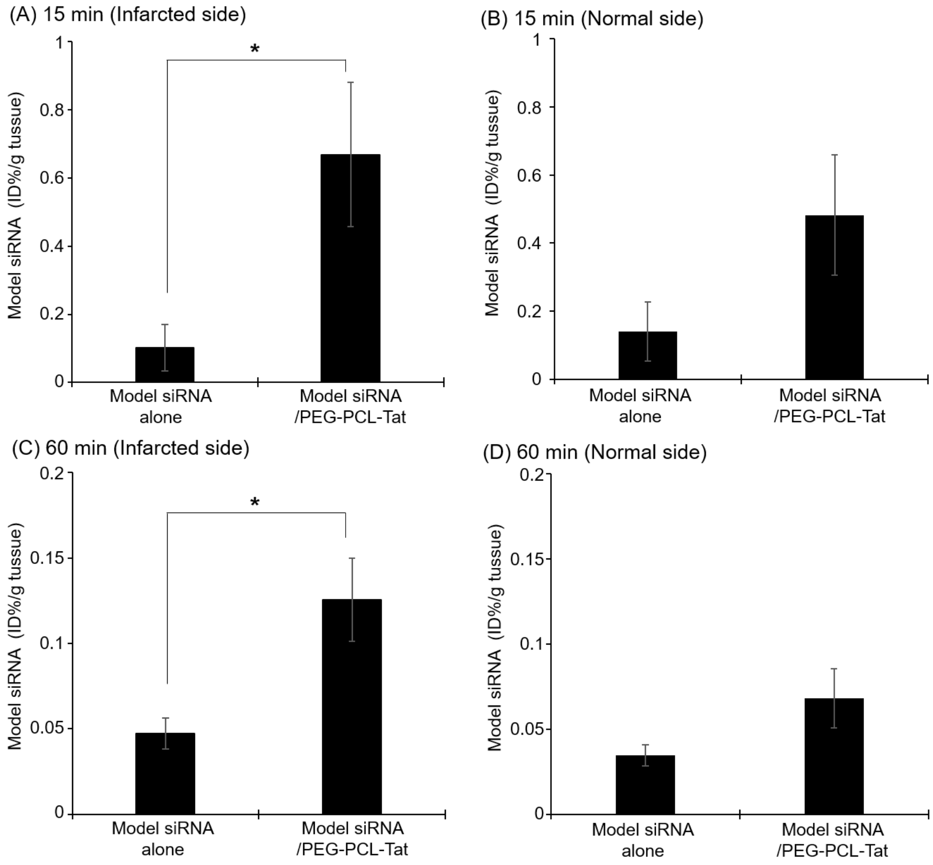

Figure S2. Brain distribution of radiolabeled model siRNA after intranasal administration, ipsilateral (infarcted side), and contralateral (normal side) forebrain in t-MCAO model mice

The percentage of administered dose per gram of brain tissue (%ID/g brain) was determined at 15 min in (A) Infarcted and (B) Normal side forebrain, and 60 min in (C) Infarcted and (D) Normal side forebrain after intranasal administration of PEG-PCL-Tat/model siRNA or model siRNA alone. Radioactivity of [^{14}C]-dextran was measured using a liquid scintillation counter and normalized to the administered dose. Data are presented as mean ± SE (n = 5). Statistical analysis was performed using an unpaired Student's *t*-test. *$p < 0.05$.
